# Quality of life in men with Klinefelter syndrome: a multicentre study

**DOI:** 10.1530/EC-23-0111

**Published:** 2023-09-19

**Authors:** Sebastian Franik, Kathrin Fleischer, Barbara Kortmann, Nike M Stikkelbroeck, Kathleen D’Hauwers, Claire Bouvattier, Jolanta Slowikowska-Hilczer, Solange Grunenwald, Tim van de Grift, Audrey Cartault, Annette Richter-Unruh, Nicole Reisch, Ute Thyen, Joanna IntHout, Hedi L Claahsen-van der Grinten, the dsd-LIFE group

**Affiliations:** 1 Department of Obstetrics and Gynaecology, Radboudumc, Nijmegen, The Netherlands; 2 Department of Pediatric Urology, Radboudumc, Nijmegen, The Netherlands; 3 Department of Internal Medicine, Radboudumc, Nijmegen, The Netherlands; 4 Department of Urology, Radboudumc, Nijmegen, The Netherlands; 5 Department for Health Evidence, Radboudumc, Nijmegen, The Netherlands; 6 Department of Pediatric Endocrinology, Bicêtre Hospital, Paris Sud University, France; 7 Department of Andrology and Reproductive Endocrinology, Medical University of Lodz, Poland; 8 Department of Endocrinology and Metabolic Disease, Centre Hospitalier Universitaire de Toulouse, France; 9 Departments of Plastic Surgery and Medical Psychology, Amsterdam UMC location VUmc, Amsterdam, The Netherlands; 10 Department of Pediatrics, Hospital Des Enfants, Toulouse, France; 11 Kinderendokrinologie und Diabetologie, Universitätsklinikum Ruhr-Universität Bochum, Kinderklinik, Bochum, Germany; 12 Department of Endocrinology, Medizinische Klinik and Poliklinik IV, University Hospital Munich, Munich, Germany; 13 Klinik fur Kinder- und Jugendmedizin, Universitat zu Lubeck, Ratzeburger Allee, Lubeck, Germany; 14 Department of Pediatric Endocrinology, Amalia Childrens Hospital, Radboudumc, Nijmegen, The Netherlands

**Keywords:** disorders/differences of sex development, Klinefelter syndrome, quality of life, multicentre study

## Abstract

**Background:**

Klinefelter syndrome (KS) is associated with an increased risk of lower socioeconomic status and a higher risk for morbidity and mortality, which may have a significant impact on quality of life (QOL). The objective of this study is to investigate QOL in a large European cohort of men with KS.

**Design:**

Cross-sectional multicentre study.

**Methods:**

Two-hundred-eighteen men with KS were recruited from 14 clinical study centres in 6 European countries which participated in the European dsd-LIFE study. Male normative data from a healthy and a psychiatric reference population were used for comparison. The validated World Health Organization (WHO) QOL (WHOQOL)-BREF questionnaire was used to investigate five main domains of quality of life (WHOQOL): global, physical, psychological, environment, and social.

**Results:**

The QOL physical domain score was lower for men with KS compared to the healthy reference population (KS: 66.9; s.d. 19.4, *n* = 193; healthy reference population: 76.5; s.d. 16.2, *n* = 1324, *P* < 0.001) but higher compared to the psychiatric reference population (54.6; s.d. 20.6; *n* = 77, *P* < 0.001). The WHOQOL-psychological domain score was lower for men with KS compared to the healthy reference population (KS: 63.6; s.d. 17.8, *n* = 193; healthy reference population: 67.8; s.d. 15.6, *n* = 1324, *P* < 0.05) but higher compared to the psychiatric reference population (45.9; s.d. 26.0), *n* = 77, *P* < 0.001). The social domain score on the WHOQOL questionnaire was found to be lower in men with Klinefelter syndrome (KS) compared to the healthy reference population (KS: 60.0; s.d. 21.6, *n* = 193; healthy reference population: 68.2; s.d. 13.8, *n* = 1324, *P* < 0.001). However, this score was similar to that of the psychiatric reference population (61.0; s.d. 17.0, *n* = 77, *P* = 0.5). The WHO environment domain score of men with KS (70.0; s.d. 15.0, *n* = 193) was similar to the healthy reference population (70.5; s.d. 20.7, *n* = 1324) but higher compared to the psychiatric reference population (61.9; s.d. 20.8, *n* = 77, *P* = 0.002). Experienced discrimination, less social activities, and the presence of chronic health problems were associated with significantly decreased QOL in men with KS.

**Conclusion:**

Overall QOL in European men with KS is significantly worse compared to a healthy European reference population. Especially, the presence of discrimination, less social activities, and chronic health problems is associated with lower physical, psychological, and social QOL. Further studies are necessary to investigate if a multidisciplinary approach may help to provide adequate counselling and psychosocial support to improve QOL.

## Introduction

With a prevalence of 1 in 660 males, Klinefelter syndrome (KS, 47,XXY) is one of the most common sex chromosome disorders (1). KS is associated with various morbidities and challenges for affected men, yet it is highly underdiagnosed most likely due to a huge variance in phenotype (1). There is a higher risk for morbidity amongst men with KS due to somatic disease and mental illness, especially cardiovascular, nervous system, endocrine, metabolic and respiratory diseases, and mental disorders such as psychoses, disorders of personality, and mental retardation (2, 3). A large study in a Danish and British cohort showed a 50% increase in mortality and a 70% increase in risk for hospital admission for men with KS (4) compared to an age-matched control group drawn randomly from the Danish civil register. A higher degree of physical impairment and lower levels of subjective general health of men with KS is also associated with a lower socioeconomic status (5). Psychosocial well-being, which included subjective well-being, self-esteem, body image, and psychological distress, was shown to be significantly inferior in a cohort of 87 patients with KS when compared to a general reference population (6). Another study in a small cohort of 43 boys with KS reported also lower psychosocial health scores, including QOL, low self-esteem, a poor self-concept, and a risk for depression (7). Men with KS might have a higher risk of experiencing discrimination due to their physical, developmental, and hormonal differences, such as the absence of sexual characteristics, reduced muscle tone, gynaecomastia, and sparse facial and body hair (1). These differences can lead to misunderstandings, stigmatization, and stereotypes about masculinity, undermining their sense of identity and self-worth. Additionally, the lack of knowledge among healthcare providers can result in limited social support, inadequate healthcare services, and challenges in accessing appropriate interventions and accommodations. Men with KS may also face discrimination due to the absence of inclusive policies, accommodations, or resources that address their unique needs.

However, little is known about the quality of life (QOL) in men with KS. Therefore, the objective of this study is to investigate QOL in a large European cohort of men with KS and to associate QOL with social activities, age at diagnosis, hormonal substitution, presence of chronic health problems, and experienced discrimination.

## Methods

### Study population

This study was part of the European dsd-LIFE study (https://www.dsd-life.eu/), a non-interventional, clinical, cross-sectional study (8). The purpose of the study was to investigate and compare the long-term outcomes of surgical and hormonal therapy and psychological and social support in adolescents and adults with different forms of disorders of sex development (DSD), aiming to provide the basis for improvements in evidence-based recommendations for care. Ethical approval was first sought from the medical ethics committee at the Charité Universitätsmedizin Berlin. Ethical approval was given by all institutional ethical boards of the participating centres, and informed consent was provided by all participants.

The dsd-LIFE consortium consists of 14 European centres in 6 European countries, that is, Germany, France, the Netherlands, Poland, Sweden, and the United Kingdom (UK). The 14 centres approached former and current patients by mail, e-mail, phone, or direct contact of the physician and promoted participation in patient support groups from February 2014 to September 2015. Participants had to be at least 16 years old with a medically confirmed clinical and/or genetic diagnosis. Details on the theoretical and methodological framework of the dsd-LIFE study have been published earlier (8).

Men with KS were asked to fill out a digital patient-reported outcome (PRO) form that comprised validated and self-constructed questionnaires on health status, mental health, QOL, psychological well-being, psychosexual outcome, testosterone treatment, fertility, experiences with care, and sexuality. To ensure confidentiality, the participants were asked to fill out the PRO with a secure password, either in the clinic or at home. Data were entered anonymously into a database.

### Reference population

We used a healthy (*n* = 1324) reference population as well as a psychiatric control group (*n* = 77) reported by Skevington *et al.*, 2012, for comparison of the mean scores for the World Health Organization QOL–BREF (WHOQOL-BREF) domain scores (9). The reference populations were recruited at 38 UK sites in community, primary care, outpatient, inpatient, rehabilitation settings, and social care. The healthy reference population included six samples of university students and student nurses, where persons with health conditions were excluded. The psychiatric population contained people with different psychiatric diagnoses, for example, depression or schizophrenia (Skevington *et al.* 2012).

### Description of outcome variables

For evaluation of QOL, a short version of the WHO-QOL-100 questionnaire, the WHOQOL-BREF questionnaire, was used. The WHOQOL-BREF questionnaire is a multiculturally validated questionnaire, evaluating QOL. The WHOQOL-BREF was developed for cross-cultural comparisons of QOL and is available in more than 40 languages, including all dsd-LIFE languages (10). Used in equal or similar cultural contexts like in-between Europe, national weightings are not needed in analyses (11). Five QOL main domains were investigated: global (two questions), physical health (seven questions), psychological (six questions), social relationships (three questions), and environment (eight questions). It is validated for persons aged 18 years and older (10). All answers are given on a 5-point Likert scale, summed per domain, and then transformed to a scale from 0 to 100 to enable comparisons between domains. Higher scores indicate a higher QOL. Domains are not scored when two or more items are missing (or one item in the three-item domain social relationship). The WHOQOL-BREF has no overall score. The domains show good psychometric properties without ceiling or floor effects and an internal consistency with Cronbach’s alpha being ≥0.8 for every domain, except for social relationships with 0.68 (10, 12).

### Possible associated factors

To ensure a homogenic KS study population, only men with a 47,XXY genotype were included in the analysis. Men with KS with a mosaicism or a different genotype (e.g. 48,XXXY) were excluded from analysis. Factors possibly associated with quality of life (QOL) that have been examined include BMI, engagement in social activities, the presence of chronic health issues, encounters with discrimination related to one's condition, encounters with discrimination for various reasons, the use of testosterone treatment, and the age at which the diagnosis was made. Further elaboration on these factors can be found in Table 1.

**Table 1:** Questions used to evaluate possible associated factors in men with KS.

Possible associated factors	Classification/question	Type	Answering options
Subjective general health	‘How is your health in general?’	ESS	Very good Good Fair Bad Very bad
Social activities	‘Compared to other people of your age, how often would you say to take part in social activities?’	ESS	Much more than most More than most About the same Less than most Much less than most
Presence of chronic health problems	‘Do you have any longstanding illness or health problem? (apart from your condition)’	SC	Yes No
Physical or mental health problem	‘Is this a physical health problem (e.g. Diabetes, coronary heart disease) or a mental health problem (e.g. depression, eating disorder)	SC	Physical health problem Mental health problem Both I don’t know
Experienced discrimination based on condition	‘Have you been discriminated against because of your condition?’	SC	Yes No
General discrimination	‘Would you describe yourself as being a member of a group that is discriminated against in this country?’ If yes: ‘On what grounds is your group discriminated against?’ ‘Colour or race, nationality, religion, language, ethnic group, age, gender, sexuality, disability, other’	ESS	Yes No
Testosterone supplement	‘Are you on testosterone therapy at present?	SC	Yes No
Age at diagnosis	‘At what age was your condition diagnosed?’	SC	Before birth At birth (0–1 month) Infancy (1 month–3 years) Childhood (4–12 years) Adolescence (13–17 years) Adulthood (≥18 years) I don’t know
Gynaecomastia	‘Presence of gynaecomastia?’	SC	Yes No
Small testes	‘Presence of small testes?’	SC	Yes No

ESS, European Social Survey question; SC, self-constructed question.

### Statistical analysis

Characteristics of the men with KS are described using means and standard deviations (s.d.) or frequencies and percentages.

Linear regression analysis was done within the KS study population to investigate possible associations between QOL and the above-described possibe associated factors, such as hormone therapy with testosterone, age at diagnosis, and presence of chronic health problems amongst men with KS. There was no correction for multiple comparisons because of the exploratory nature of this study and the primary concern about type II error. Domain scores of the WHOQOL-BREF for men with KS, the healthy, and the psychiatric reference population have been compared by unpaired *t*-tests. Furthermore, a network plot for showing associations between QOL and variables of possible influence was created using Pearson correlation coefficients, restricted to correlation coefficients with a *P*-value < 0.05. The network plot was created with the SemiPar package (https://CRAN.R-project.org/package=SemiPar) using the statistical software R version 4.2.1 (https://www.r-project.org/). For all other analyses, SPSS software version 22.0 was used (IBM Corp. Released 2013. IBM SPSS Statistics for Windows, Version 22.0. Armonk, NY: IBM Corp).

## Results

### Basic characteristics of the KS study population

A total of 218 men with KS were included in the study, but thirteen men with KS were excluded from the analysis due to mosaicism or more than one additional X-chromosome. A total of 205 men with KS had a 47,XXY karyotype and were included in the analysis. The baseline characteristics of men with KS are listed in Table 2. KS was diagnosed in 11/205 (5%) men prenatally, in 52/205 (25%) men during childhood/adolescence, and in 120/205 (59%) during adulthood; for the remaining 22/205 (11%) men with KS, age at diagnosis was unknown. Discrimination based on KS was reported to be experienced in 20.5% of men with KS in our study cohort. Discrimination based on other reasons was also investigated, showing very low percentages of discrimination based on ethnicity, language, colour, or race (0.5% each) and also low percentages for sexuality (3.2%) or disability (3.2%) (Table 2).

**Table 2:** Baseline characteristics of men with Klinefelter syndrome.

	Participants with Klinefelter syndrome (*n* = 205 )
Age in years, mean (s.d.), range	39.9 (15.0), 15–75
Height in cm, mean (s.d.)	184.0 (12.5)
Weight in kg, mean (s.d.)	82.6 (27.6)
BMI in kg/m^2^, mean (s.d.)	24.6 (6.7)
Country of residence, *n* (%)	
Germany	36 (17.6%)
France	23 (11.2%)
Netherlands	83 (40.5%)
Poland	23 (11.2%)
Sweden	32 (15.6%)
United Kingdom	8 (3.9%)
Testosterone supplement at present, *n* (%)	
Yes	145 (70.7%)
No	9 (4.4%)
Unknown	51 (24.9%)
Age at diagnosis, *n* (%)	
Prenatal	11 (5.4%)
Childhood	52 (25.4%)
Adulthood	120 (58.5%)
Unknown	22 (10.8%)
Social activities, *n* (%)	
Much more than most	5 (2.4%)
More than most	17 (8.3%)
About the same	74 (36.1%)
Less than most	61 (29.8%)
Much less than most	26 (12.7%)
Unknown	22 (10.7%)
Presence of chronic health problems, *n* (%)	
Yes	109 (53.2%)
No	69 (33.7%)
Unknown	27 (13.2%)
Experienced discrimination based on condition, *n* (%)	
Yes	42 (20.5%)
No	139 (67.8%)
Unknown	24 (11.7%)
Member of a group discriminated against, based on (*n*(%))	
Colour or race	1 (0.5%)
Language	1 (0.5%)
Ethnic group	1 (0.5%)
Age	1 (0.5%)
Gender	4 (1.8%)
Sexuality	7 (3.2%)
Disability	7 (3.2%)
Other	1 (0.5%)
Subjective general health, *n* (%)	
Very good	18 (8.8%)
Good	86 (42.0%)
Fair	59 (28.8%)
Bad	23 (11.2%)
Very bad	5 (2.4%)
Unknown	14 (6.8%)
Gynaecomastia, *n* (%)	
Yes	36 (17.6%)
No	169 (82.4%)
Small testes, *n* (%)	
Yes	83 (40.5%)
No	122 (59.5%)

### WHOQOL-BREF in men with KS and reference populations

#### Quality of life global was lower (−15.7) amongst participants who experienced discrimination

**Figure 1:**
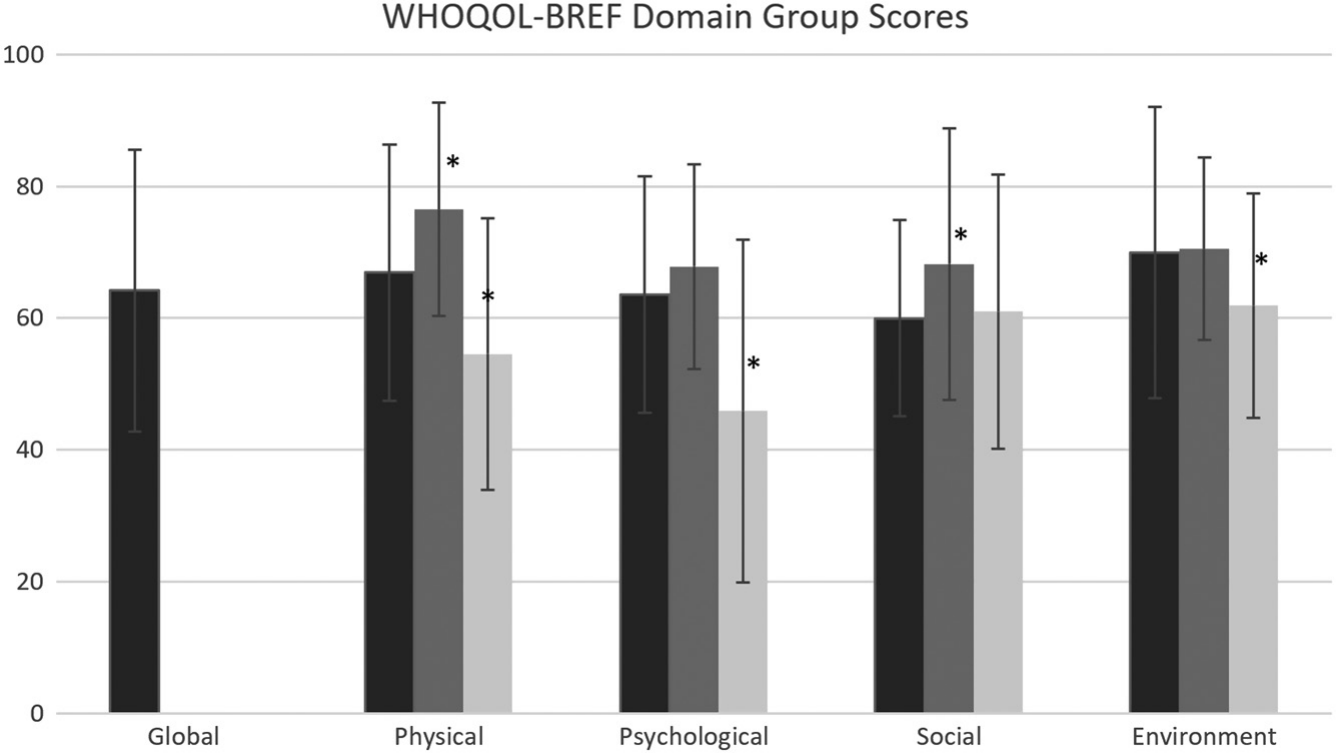
WHOQOL-BREF domain scores (*Y*-axis, 0–100) and s.d. (errorbars) for men with Klinefelter syndrome from the dsd-LIFE study (black, *n* = 193), for the healthy reference population (grey, *n* = 1324) and the psychiatric reference population (light grey, *n* = 77). *P < 0.01, *P*-values showing statistically significant differences between men with KS and the respective reference population based on unpaired two-tailed *t*-test.

**Figure 2:**
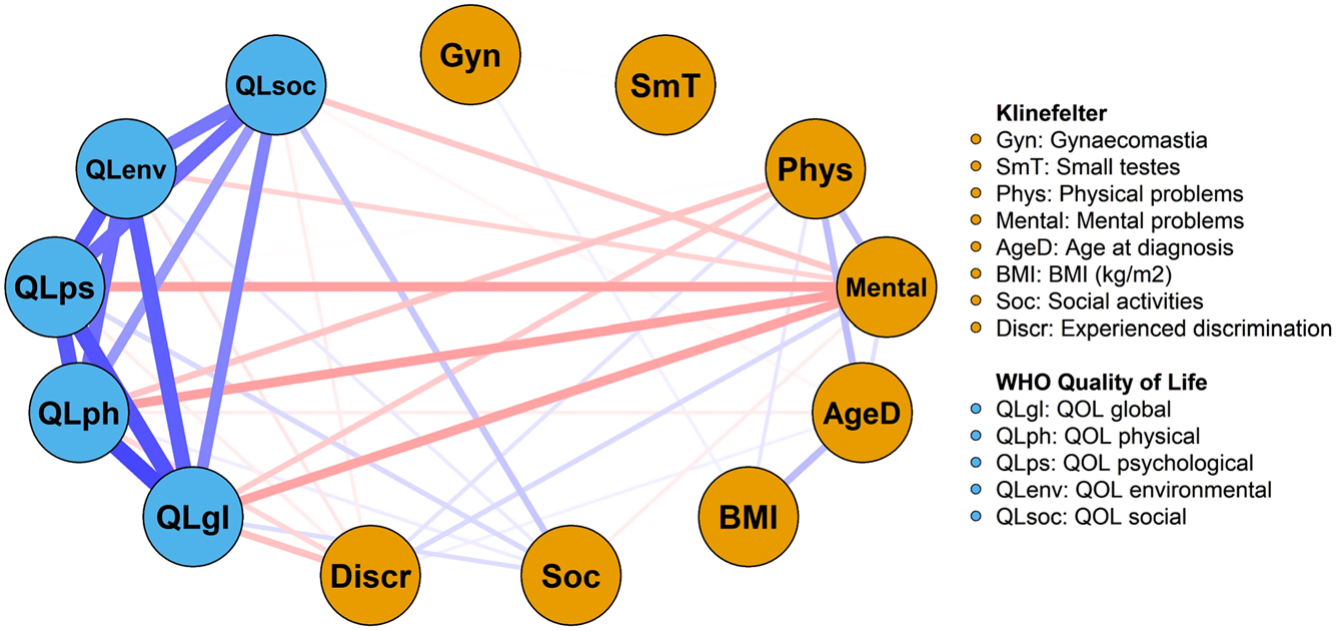
Network plot visualizing Pearson correlation coefficients (*R*) for various variables of men with KS (*n* = 193). Blue lines represent positive associations, and red lines negative associations. A stronger (thicker, darker) line indicates a stronger association (*R*) between two variables. For visual clarity, only associations with a *P*-value <0.05 are presented.

The average WHOQOL-BREF global was completed by 193 men with KS and the mean group score was 64.2 (s.d. 21.7) (Fig. 1). The score amongst participants who reported to have experienced discrimination based on their condition was significantly lower (−15.7, CI −22.7; −8.7) compared to participants who did not experience discrimination. Furthermore, the patient-reported presence of chronic health problems resulted in statistically significantly lower QOL (mean score 57.3, s.d. 23.4) compared to participants without the presence of chronic health problems (mean score 72.5, s.d. 15.0). Especially, mental chronic health problems were associated with a lower QOL (mean score 45.8, s.d. 23.3), compared to the presence of physical chronic health problems for men with KS (mean score 61.4, s.d. 21.5). Furthermore, less participation in social activities was associated with a lower global group score for QOL (Table 3). There was no significant association between global QOL and BMI (*P* = 0.45), current testosterone therapy (*P* = 0.46), and age at diagnosis (*P* = 0.94). Our network plot shows strong positive associations between the QOL global domain and all other QOL domains (Fig. 2).

**Table 3:** Results of linear regression analysis of the WHOQOL-BREF domain scores versus possible associated factors in our study population of men with Klinefelter syndrome (*n* = 193).

	Independent variable	Regression coefficient (B)	95% CI	*P*-value
WHOQOL-BREF Global Group score (scale: 1–100)	Participation in social activities (yes/no)	4.1	0.9; 7.4	0.01
	Age at diagnosis (years)	−3.8	−12.1; 4.5	0.94
	Testosterone substitution (yes/no)	−1.2	−3.3; 0.9	0.46
	Presence of chronic health problems (yes/no)	−14.1	−20.8; −7.3	<0.01
	Experienced discrimination (yes/no)	−15.7	−22.7; −8.7	<0.01
	BMI (kg/m^2^)	−0.1	−0.9; −0.7	0.45
WHOQOL-BREF physical domain score (scale: 1–100)	Participation in social activities	3.0	0.03; 5.9	0.05
	Age at diagnosis	−5.5	−13.3; 2.2	0.77
	Testosterone substitution	2.0	−11.6; 15.6	0.83
	Presence of chronic health problems	−13.3	−19.3; −7.4	<0.01
	Experienced discrimination	−12.2	−18.6; −5.7	<0.01
	BMI	0.1	−0.5; 0.8	0.22
WHOQOL-BREF psychological domain score (scale: 1–100)	Participation in social activities	4.5	1.8; 7.2	0.01
	Age at diagnosis	−1.6	−9.0; 5.8	0.37
	Testosterone substitution	−7.2	−20.1; 5.6	0.06
	Presence of chronic health problems	−6.2	−12.1; −0.3	0.04
	Experienced discrimination	−7.7	−13.9; −1.6	0.01
	BMI	−0.2	−0.9; 0.5	0.10
WHOQOL-BREF social domain score (scale: 1–100)	Participation in social activities	7.3	4.0; 10.5	<0.01
	Age at diagnosis	−3.2	−12.6; 6.1	0.35
	Testosterone substitution	−9.9	−25.9; 6.0	0.15
	Presence of chronic health problems	−3.6	−10.8; 3.6	0.26
	Experienced discrimination	−10.5	−18.2; −2.8	<0.01
	BMI	−0.01	−0.9; 0.9	0.75
WHOQOL-BREF environment score (scale: 1–100)	Participation in social activities	2.3	0.03; 4.5	0.05
	Age at diagnosis	−1.8	−7.8; 4.3	0.41
	Testosterone substitution	−2.1	−12.0; 7.7	0.53
	Presence of chronic health problems	−5.8	−10.5; −1.1	0.01
	Experienced discrimination	−8.4	−13.3; −3.4	<0.01
	BMI	0.1	−0.5; 0.6	0.64

#### Quality of life physical domain was lower (66.9) compared to the healthy reference population (76.5)

The mean WHOQOL-physical domain score of men with KS (*n* = 193) was 66.9 (Fig. 1). This was significantly lower compared to the healthy reference population (*n* = 1324) who achieved a mean score of 76.5 (*P* < 0.001, Fig. 1). However, the reference population with psychiatric illness (*n* = 77) scored significantly lower (mean score 54.6) than men with KS (*P* < 0.001) and the healthy reference population (*P* < 0.001, Fig. 1).

Our univariate analysis shows that the mean QOL physical domain score was significantly lower amongst men with KS who reported to have experienced discrimination based on their KS condition (mean score 57.1) compared to men who did not experience discrimination (mean score 69.0). Furthermore, men with the patient-reported presence of chronic health problems had a significantly lower QOL physical domain score (mean score 60.5) compared to men without the presence of chronic health problems (mean score 75.3). Less participation in social activities was also associated with lower physical domain scores. There was no significant association between the QOL physical domain score and current testosterone therapy (*P* = 0.83), BMI (*P* = 0.22), or age at diagnosis (*P* = 0.77).

#### Quality of life psychological health domain was lower (63.6) compared to the healthy reference population (67.8)

The mean WHOQOL-psychological health domain score of men with KS (*n* = 193) was 63.6 (s.d. 17.8). This was significantly lower compared to the healthy reference population (*n* = 1324) who had a mean score of 67.8 (*P* < 0.001 Fig. 1). However, the reference population with psychiatric illness (*n* = 77) scored significantly lower than the men with KS and the healthy reference population with a mean score of 45.9 (*P* < 0.001; Fig. 1). Our univariate analysis shows that the mean psychological health domain score was significantly lower amongst men with KS who reported to have experienced discrimination based on their condition (mean score 56.6) compared to men who did not (mean score 65.0; Table 3). Furthermore, men with the patient-reported presence of chronic health problems had significantly lower QOL (mean score 60.2) compared to men without the presence of chronic health problems (mean score 67.4; Table 3). Less participation in social activities was also associated with lower psychological domain scores (Table 3). There was no significant association with current testosterone therapy (*P* = 0.06), BMI (*P* = 0.10), or age at diagnosis (*P* = 0.37) (Fig. 2).

#### Quality of life social domain was lower (60.0) compared to the healthy reference population (68.2)

The mean WHOQOL-social domain score of men with KS (*n* = 193) was 60.0. This was significantly lower compared to the healthy reference population (*n* = 1324) who achieved a mean score of 68.2 (*P* < 0.001, Fig. 1). The WHOQOL-social domain score of men with KS was similar to the reference population with psychiatric illness (*n* = 77) who had a mean score of 61.0 (*P* = 0.5, Fig. 1). The mean social domain score was significantly lower amongst men with KS who reported to have experienced discrimination based on their condition (50.4) compared to men who have not experienced discrimination (mean score 61.9, *P* = 0.008). Less participation in social activities was associated with lower social domain scores (*P* < 0.001). There were no statistically significant associations with self-reported presence of chronic health problems (*P* = 0.26), BMI (*P* = 0.75), testosterone therapy at present (*P* = 0.15), or age at diagnosis (*P* = 0.35).

#### Quality of life environment domain was similar (70.0) to the healthy reference population (70.5)

The WHOQOL-environment domain score of men with KS (*n* = 193) was 70.0. This was comparable to the healthy reference population (*n* = 1324) who achieved a mean score of 70.5 (*P* = 0.5, Fig. 1). The reference population with psychiatric illness (mean score 61.9, *n* = 77) scored significantly lower than the men with KS (*P* = 0.002) and the healthy reference population (*P* < 0.001); Fig. 1). The mean environment domain score was significantly lower amongst men with KS who reported to have experienced discrimination based on their condition (mean score 64.2) compared to men who have not experienced discrimination (mean score 71.4; Table 3). Furthermore, the patient-reported presence of chronic health problems was associated with lower scores for WHOQOL-environment (mean score 68.2) compared to men without the presence of chronic health problems (mean score 73.4; Table 3). Less participation in social activities was also associated with lower environment domain scores (*P* < 0.047; Table 3). There were no significant associations with testosterone therapy at present (*P* = 0.53), BMI (*P* = 0.64), or age at diagnosis (*P* = 0.41).

## Discussion

This is the first large European multicentre study comparing QOL in a large group of men with KS with a healthy UK reference population and a psychiatric reference population. Our study has shown that global QOL in men with KS is significantly lower compared to a UK reference population. The global QOL is lowest amongst those persons with KS who had experienced discrimination during their life or suffer from chronic mental health problems. It is important to promote early support and inclusivity to enhance the QOL for individuals with KS. This is supported by a previous, smaller study investigating QOL in 43 adolescents with KS, reporting that a poor outcome in QOL directly correlated to the severity of the phenotype, measured as a composite score of physical traits including tall stature, eunuchoid body proportion, wide arm span, large waist circumference, high BMI, small testicular volume, short phallus, or gynaecomastia (7). Furthermore, lower scores compared to a reference population for QOL, self-esteem, body image, and mental health were reported in a study using validated questionnaires like the ‘Personal Wellbeing Index’ and the ‘Rosenberg Self-esteem Scale’ among 87 adult men with KS (6). Herlihy *et al.* have also shown that the age of diagnosis was not a predictor for the presence of a more severe phenotype of KS. In that study, the phenotype was measured as a composite score of variables such as testosterone deficiency, breast development, infertility, physical development and learning, behavioural, and communication difficulties. In accordance with these results, there was no significant association between the age of diagnosis and QOL in our study population. There was also no significant association between QOL and testosterone supplementation at present in our study. This result should be interpreted with caution because 145 of 205 participants had testosterone supplementation and only 9 men did not take testosterone supplementation; for the remaining participants, it was unknown. However, our findings were confirmed by another study, investigating QOL in 132 men with KS in Denmark (13). In their study, there was also no significant difference between the two KS subgroups with or without testosterone therapy (13). They also found that men with KS scored significantly lower for both physical and psychological QOL, compared to a matched Danish cohort from the registry. This was confirmed in our study; men with KS scored a significantly lower QOL in the physical, psychological, and social domains compared to the healthy European reference population. This may be explained not only by the presence of chronic health problems, such as psychoses, disorders of personality, and mental retardation, and problems with participation in social activities (2) but also by the fact that men with KS have a higher risk for chronic diseases such as diabetes or heart diseases, which are also associated with lower scores for QOL (2, 14, 15). Compared to a European reference population with a diagnosis of various psychiatric illnesses such as depression and schizophrenia, the scores were higher for men with KS in the physical, psychological, and environmental QOL domains but not in the social domain. This finding confirms earlier studies indicating that participation in social activities often remains challenging for men with KS (5, 16). Additionally, the lower QOL in the psychological health domain may be associated with the finding that depression and anxiety are often present in men with KS (16). Potentially contributing factors such as bullying, lower self-esteem issues, and social challenges should carefully be evaluated by healthcare providers (17, 18, 19).

A novel finding of this study is that men with KS reported less participation in social activities compared to the healthy European reference population and reported lower scores in the QOL social domain. Their overall QOL social domain scores were even lower when discrimination based on condition was experienced. Several studies reported higher levels of distress during social interactions, shyness, and social anxiety and withdrawal amongst men with KS (20, 21, 22). This may result in less (satisfactory) social activities. In order to improve social skills, men with KS may benefit from early social training and training in coping skills. The need for early social support is emphasized by a study showing that employment status and social support are, amongst others, the best predictors of psychosocial well-being (6). Therefore, social engagement and involvement in activities could play a role in enhancing the QOL for individuals with KS. Promoting social inclusion and providing opportunities for social participation may contribute to improved well-being and reduced discrimination.

### Limitations

This is the largest study of men with KS using a validated questionnaire to investigate QOL. There was no matched reference group for our cohort of men with KS; therefore, we used published data from a general European reference population with a mean age of the reference population was comparable to our study group. Unfortunately, almost all cases of the control group were from the UK and their BMI was unknown, which might be a confounder in the study. A main limitation of this study is the possibility of selection bias, as men with KS were mostly recruited from participating specialized outpatient clinics and from patient support groups (8). Unfortunately, it is unknown how many possible participants have been contacted at the different recruiting clinics and patient support groups but were not willing to participate. Furthermore, the questionnaire used in this study was rather long, taking about 3 h to fill in, which has led to incompletely filled-in questionnaires and more than 10% missing outcomes for some variables (attrition bias). Furthermore, many questions were dichotomous with ‘yes’ and ‘no’ as possible answers. Another limitation of this study is its retrospective, explorative design and that parts of the questionnaire contained self-constructed questions which were not validated. Furthermore, some medical information such as small testes, gynaecomastia, and testosterone treatment have been collected using a patient-reported survey, which can affect the accuracy of the outcomes. In the linear regression analysis, there was no adjustment for multiple comparisons because of the exploratory nature of this study and the primary concern about type II error.

## Conclusion

Overall QOL in European men with KS is significantly inferior compared to a healthy European reference population. Especially, the presence of discrimination, less social activities, and chronic health problems are associated with lower global, physical, psychological, and social QOL. Further intervention studies are necessary to investigate if a multidisciplinary approach may help to provide adequate counselling and psychosocial support to improve the QOL of men with KS.

## Declaration of interest

All authors declare no support from any organization for the submitted work; no relationship with any organizations that might have an interest in the submitted work in the previous three years; no other relationships or activities that could appear to have influenced the submitted work. Therefore, the authors declare that they have no competing interests.

## Funding

The study leading to these results has received funding from the European Union’s Seventh Framework Programme (FP7/2007–2013) under grant agreement n°305373.

## Trial registration

German Clinical Trials Register: Registration identification number: DRKS00006072, date of registration April 17th, 2014. DRKS00006072 (German Clinical Trials Register).

## Availability of data and materials

The datasets analysed during the current study are not publicly available as long as primary analyses for other outcomes of dsd-LIFE are not completed. Afterwards, scientific public use files are planned. The data will be made available by the principal investigator upon request to researchers after publication of the primary outcomes described in the grant by the consortium.
